# Inflammation Impacts Androgen Receptor Signaling in Basal Prostate Stem Cells Through Interleukin 1 Receptor Antagonist

**DOI:** 10.21203/rs.3.rs-3539806/v1

**Published:** 2023-12-15

**Authors:** Paula O. Cooper, Jiang Yang, Hsing-Hui Wang, Meaghan M. Broman, Gada D. Awdalkreem, Gregory M. Cresswell, Liang Wang, Emery Goossens, Nadia A. Lanman, Rebecca W. Doerge, Faye Zheng, Liang Cheng, Scott A. Crist, Robert E. Braun, Travis J. Jerde, Timothy L. Ratliff

**Affiliations:** 1 Department of Comparative Pathobiology, College of Veterinary Medicine, Purdue University, West Lafayette, IN 47907, USA; 2 Purdue Institute for Cancer Research, West Lafayette, IN 47907, USA; 3 Department of Pharmacology and Toxicology, Department of Urology, Department of Microbiology and Immunology, Indiana University School of Medicine, Indianapolis, IN 46202, USA; 4 Department of Statistics, Purdue University, West Lafayette, IN 47907, USA; 5 Department of Pathology and Laboratory Medicine, Indiana University School of Medicine, Indianapolis, IN 46202, USA; 6 The Jackson Laboratory, Bar Harbor, ME 04609, USA; 7 These authors contributed equally to the manuscript.

**Keywords:** prostate, stem cells, inflammation, androgen receptor, interleukin 1 receptor antagonist

## Abstract

The majority of patients with benign prostate hyperplasia (BPH) exhibit chronic prostate inflammation and the extent of inflammation correlates with the severity of symptoms. How inflammation contributes to prostate enlargement and/or BPH symptoms and the underlying mechanisms are not clearly understood. We established a unique mouse model Prostate Ovalbumin Expressing Transgenic 3 (POET3) that mimics chronic non-bacterial prostatitis in men to study the role of inflammation in prostate hyperplasia. After the injection of ovalbumin peptide-specific T cells, POET3 prostates exhibited an influx of inflammatory cells and an increase in pro-inflammatory cytokines that led to epithelial and stromal hyperplasia. We have previously demonstrated with the POET3 model that inflammation expands the basal prostate stem cell (bPSC) population and promotes bPSC differentiation in organoid cultures. In this study, we investigated the mechanisms underlying the impact of inflammation on bPSC. We found that AR activity was enhanced in inflamed bPSC and was essential for bPSC differentiation in organoid cultures. Most importantly, we identified, for the first time, interleukin 1 receptor antagonist (IL-1RA) as a key regulator of AR in basal stem cells. IL-1RA was one of the top genes upregulated by inflammation and inhibition of IL-1RA abrogated the enhanced AR nuclear accumulation and activity in organoids derived from inflamed bPSC. The mirroring effects of IL-1RA recombinant protein and IL-1α neutralizing antibody suggest that IL-1RA may function by antagonizing IL-1α inhibition of AR expression. Furthermore, we established a lineage tracing model to follow bPSC during inflammation and under castrate conditions. We found that inflammation induced bPSC proliferation and differentiation into luminal cells even under castrate conditions, indicating that AR activation driven by inflammation in bPSC is sufficient for their proliferation and differentiation under androgen-deprived conditions. However, proliferation of the differentiated bPSC in the luminal layer significantly diminished with castration, suggesting inflammation may not maintain AR activity in stromal cells, as stromal cells deprived of androgen after castration could no longer provide paracrine growth factors essential for luminal proliferation. Taken together, we have discovered novel mechanisms through which inflammation modulates AR signaling in bPSC and induces bPSC luminal differentiation that contributes to prostate hyperplasia.

## Introduction

The coexistence of inflammation and benign prostate hyperplasia (BPH) are well documented^1, 2^. The majority of BPH patients exhibit chronic inflammation and the extent of inflammation correlates with the severity of symptoms^3, 4^. However, a direct link between inflammation and BPH remains an open question. Inflammation may contribute to BPH through multiple mechanisms^2^. One important mechanism may be through its modulation of prostate stem cell populations, which have long been implicated in hyperproliferative diseases^5, 6^.

Stem cells within the prostate basal epithelium (basal prostate stem cells, bPSC) can differentiate into both luminal and basal lineages in *in vitro* organoid cultures and have been shown to contribute to the repair and recovery of the luminal epithelium in various animal models for prostate diseases and injuries. For example, lineage tracing models tracking bPSC have shown that these stem cells contribute to the repair of prostate luminal epithelium in an induced luminal anoikis model^7^, or to a smaller extent, during the castration-regeneration cycles^8, 9^. We and others have shown that inflammation greatly impacts the behavior of bPSC. In our previous studies, we established a unique mouse model (Prostate Ovalbumin Expressing Transgenic 3 (POET3)) that closely models human chronic non-bacterial prostatitis, the most commonly diagnosed clinical prostatitis^10^. After adoptive transfer of pre-activated ovalbumin peptide-specific CD8+ T cells isolated from the spleens of OT-1 transgenic mice, this model experiences an influx of inflammatory immune cells into the prostate, including CD8+, CD4+, and Foxp3+/CD4+ T cells as well as myeloid-derived suppressor cells (MDSC), and an increase in pro-inflammatory cytokine production^10^, which leads to the development of epithelial and stromal hyperplasia. Along with the induction of inflammation, we observed a significant expansion of bPSC population, which produced larger and more differentiated organoids (resembling type II tubule-like organoids, based on classifications by Kwon *et al.*^11^) in a 3D androgen-free culture system^12^. Inflammation-induced expansion of the bPSC population and enhanced bPSC differentiation capability were also described in other inflammation models, including bacteria-induced acute prostatitis^13, 14^. The mechanisms underlying the impact of inflammation on bPSC and how the bPSC contributes to prostate hyperplasia are still poorly understood.

The androgen receptor (AR) plays a critical role in prostate homeostasis and maintenance. Drugs that target androgen synthesis and signaling remain as important treatments for both benign and malignant prostate diseases. In addition to its strong presence in the epithelium, AR activation in prostate stromal cells releases paracrine growth factors (e.g. NRG, FGF, IGF, EGF), which act on the corresponding receptors on luminal epithelial cells and contribute to epithelial and stromal expansion and the regeneration of luminal epithelium during the castration-regeneration cycle^8, 9^. While strong AR nuclear presence is prevalent in luminal epithelial cells, AR staining is observed in about half of total basal epithelial cells^8^. Not surprisingly, AR is dispensable in the regression and recovery of basal epithelium during the castration and regeneration process^8^. However, AR is required for the differentiation of bPSC into luminal epithelial cells during the castration-regeneration cycle, as the number of lineage-traced bPSC in the luminal layer during regeneration was significantly reduced in basal epithelium-specific AR knockout mice^8^.

In the current study, we determined that both AR expression and activity were markedly stimulated in bPSC isolated from inflamed prostates in our POET3/OT-1 prostatitis model. With both organoid cultures and *in vivo* lineage tracing models, we have shown that AR signaling is essential for the differentiation of bPSC into luminal epithelial cells during prostatitis and inflammation stimulates AR even under androgen-deprived castrate conditions, which is sufficient to trigger basal to luminal differentiation. Most importantly, we identified a novel mechanism for AR modulation in bPSC where interleukin-1 receptor antagonist (IL-1RA) expressed by bPSC was shown to enhance nuclear localization of AR, modulate AR-dependent gene expression and function as the driver for bPSC luminal differentiation.

## Results

### AR activity is elevated in prostate basal stem cells isolated from inflamed POET3 mice

**Figure 1.**
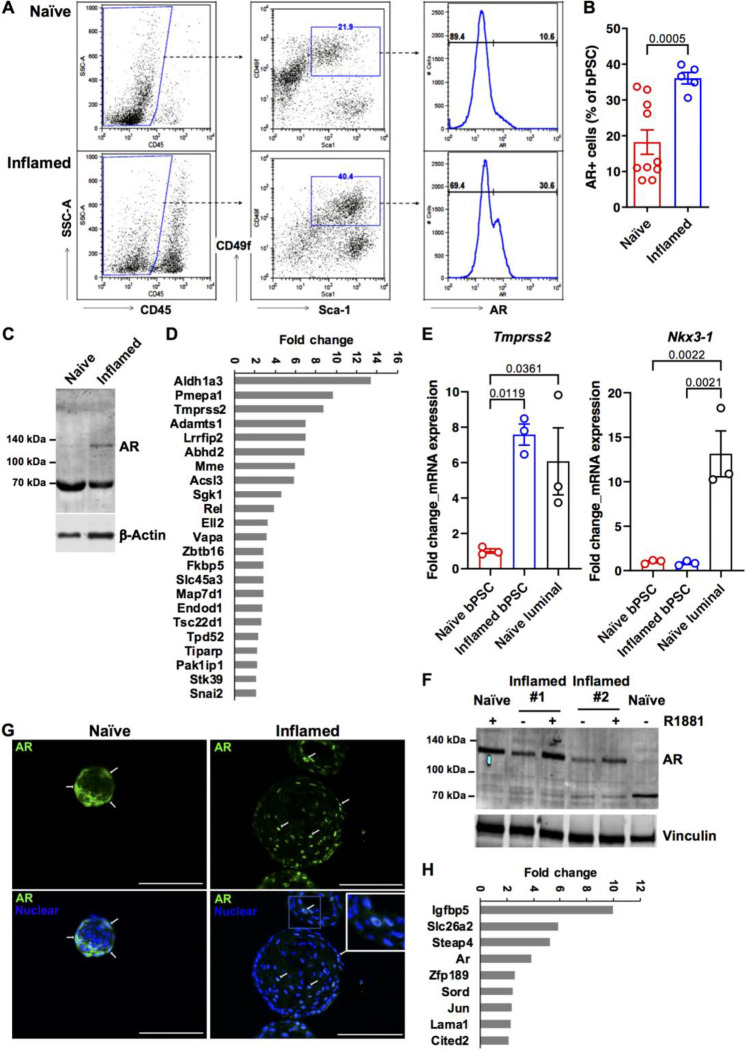
AR expression and activity are elevated in bPSC freshly isolated from inflamed prostates and the enhanced AR activity was sustained in inflamed organoids in the absence of androgen. (**A**) Representative flow cytometry dot plots and histograms of intracellular AR in bPSC from naïve and inflamed prostates. (**B**) Bar graph showing the percentage of AR+ cells within naïve and inflamed bPSC population as analyzed with flow cytometry (Naïve, n=10; Inflamed, n=5). (**C**) Immunoblot of full-length AR in naïve and inflamed bPSC lysates. (**D**) Fold change of AR target genes significantly upregulated in inflamed bPSC (Naïve or Inflamed, n=3). (**E**) qRT-PCR of conventional AR target genes, *Tmprss2* and *Nkx3–1,* in bPSC. Matched luminal population is included as a control (Naïve or Inflamed, n=3). (**F**) Immunoblot showing increased level of full-length AR in inflamed organoids compared to naïve organoids. Full-length AR levels in both naïve and inflamed organoids were elevated with 1 nM R1881 treatment. (**G**) AR Immunofluorescence staining in organoids derived from naïve or inflamed bPSC. Representative cells with nuclear localized AR are indicated with white arrows AR nuclear localization is also shown in the inset image with higher magnification (Scale bar, 100 μm). (**H**) Fold change of AR target genes significantly upregulated in inflamed organoids (Naïve or Inflamed, n=3).

Given the critical function of AR in prostate homeostasis and stem cell differentiation, we assessed the impact of inflammation in our POET3/OT-1 prostatitis model on AR signaling in bPSC population. As shown in Fig. 1A, bPSC were identified via flow cytometry as CD45−/CD31−, Sca-1+ and CD49f+ (middle panels). A significantly higher percentage of the inflamed bPSC was positive for full-length AR identified with an antibody against C-terminal AR (Fig. 1A, right panels, and 1B). Consistent with the flow cytometry data, immunoblot using an antibody against N-terminal AR detected an AR band at about 120 kDa in inflamed bPSC, while, consistent with previous characterization studies^15^, the naïve bPSC produced a strong band of degraded AR at about 70 kDa (Fig. 1C). To verify increased AR activity, a qRT-PCR array for AR signaling target genes was used to compare the naïve and inflamed bPSC samples. The resulting heatmap illustrates the differential expression of various AR target genes between naïve and inflamed bPSC (Supplemental Fig. 1A), with twenty-three genes, including *Tmprss2*, significantly upregulated in inflamed bPSC (Fig. 1D, Supplemental Table 1). The upregulation of *Tmprss2* in inflamed bPSC was further verified with qRT-PCR and its level in inflamed bPSC was comparable with the level observed in luminal epithelial cells (Fig. 1E). We further examined other canonical luminal AR target genes via qRT-PCR and found that the canonical target *Nkx3–1* remained at similar levels between naïve and inflamed bPSC (Fig. 1E). Therefore, these data demonstrate that both AR levels and activity were increased in inflamed bPSC and AR activation driven by inflammation may trigger a different target gene program.

### Inflammation-driven AR activity is sustained in bPSC-derived organoids under androgen-deficient condition and is essential for the organoid growth and differentiation

To examine the persistence of altered AR signaling upon removal from the inflammatory environment and circulating androgens, bPSC were isolated from naïve or inflamed prostates and cultured under defined and serum-free organoid culture conditions in the absence of androgen^12^. AR expression and activity were analyzed in formed organoids. Immunoblot of lysates from naïve or inflamed organoids revealed that full-length AR was elevated in inflamed organoids and its levels were further increased with synthetic AR ligand R1881 (Fig. 1F). R1881 was able to stabilize AR in the naïve organoids, with band shifting from the degraded product at 70 kDa to full-length AR at 120 kDa^15^ (Fig. 1F). Consistent with the increase in total AR protein level, a significantly higher percentage of cells with overall or nuclear AR staining was observed in organoids derived from inflamed bPSC compared to those from naïve bPSC (Fig. 1G, with quantitation in Fig. 4C, Naïve_control and Inflamed_control groups), indicating that AR remains active in inflamed organoids even in the absence of androgen and inflammatory cells. Compared to the PCR array data collected from freshly isolated bPSC (Fig. 1D), AR target gene array with the organoids identified a smaller, yet intriguing set of significantly upregulated AR responsive genes in the inflamed organoids (Fig. 1H, Supplemental Fig. 1B, Supplemental Table 2). The modulation of these target genes confirmed the enhanced AR activity in organoids derived from inflamed bPSC. Furthermore, the difference in AR-responsive genes between freshly isolated bPSC and organoids suggests that AR may perform distinct functions in organoid growth and differentiation in androgen-depleted conditions.

**Figure 2.**
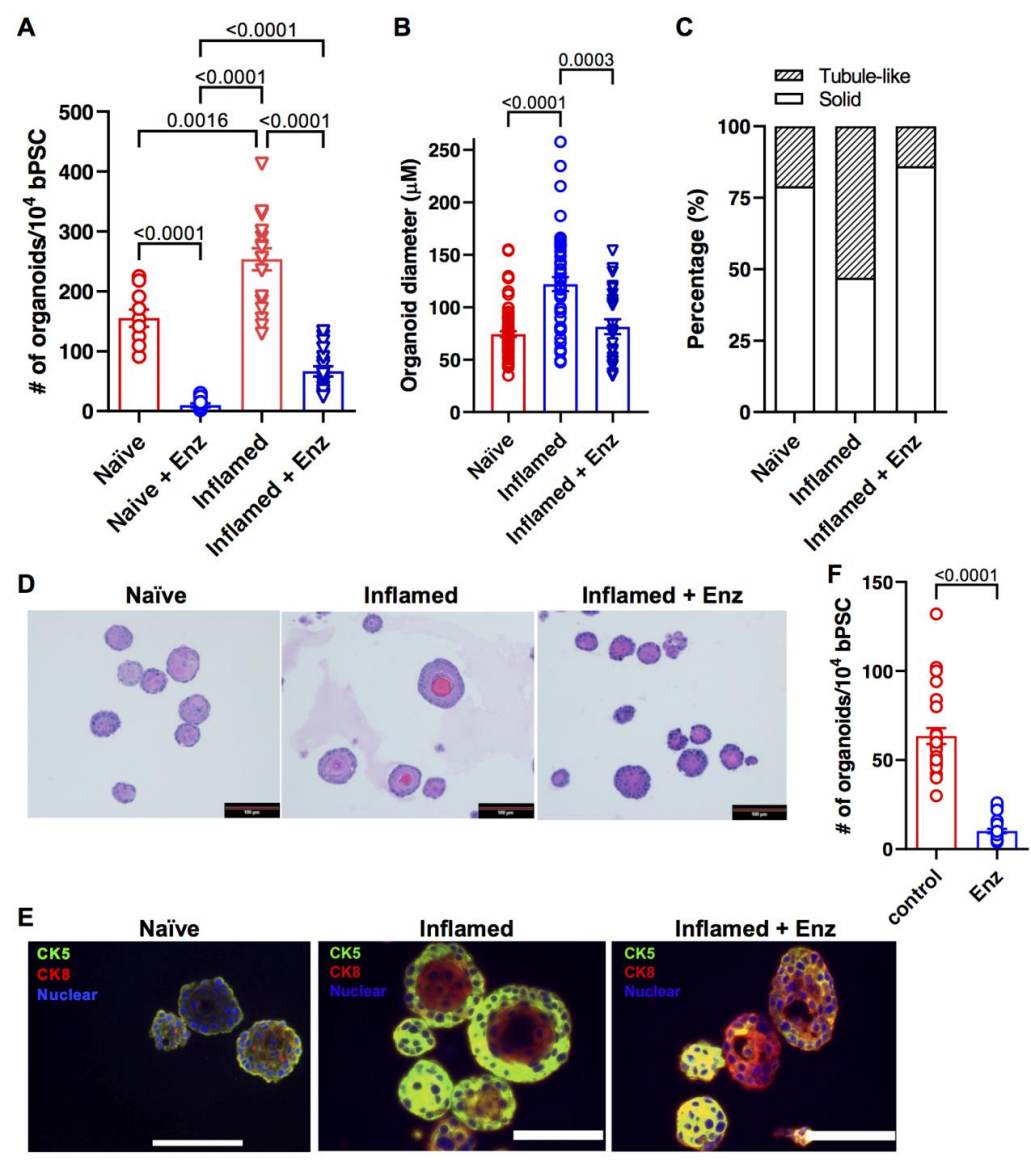
Abrogation of AR activity inhibits the differentiation of inflamed bPSC in organoid cultures. (**A**) Number of organoids formed with naïve, inflamed bPSC with or without 10 μM Enz treatment (Naïve groups, n=10; Inflamed groups, n=18). **(B)** Diameter of organoids and **(C)** percentage of organoids with tubule-like structures in naïve, inflamed organoids or inflamed organoids treated with Enz (Naïve, n=74; Inflamed, n=47; Inflamed+Enz, n=25). **(D)** Representative H&E images of naïve, inflamed organoids or inflamed organoids treated with Enz (Scale bar, 100 μm). **(E)** CK5 (green) and CK8 (red) staining showing lack of organization in Enz-treated inflamed organoids (Scale bar, 25 μm). **(F)** Enz significantly reduces the number of organoids formed with human PSC (control or Enz, n=27).

Given the strong expression of full-length AR in the inflamed bPSC and organoids, coupled with induced AR target gene expression and increased bPSC differentiation, we reasoned that the effects inflammation exerted on bPSC may be mediated through AR. To verify the requirement for AR signaling in inflammation-driven organoid proliferation and differentiation, naïve or inflamed bPSC were treated with the clinical AR antagonist, Enzalutamide (Enz), for the duration of organoid culture. AR inhibition resulted in a significant 74% reduction in inflamed organoid formation and 94% reduction in naïve organoid formation (Fig. 2A). These data suggest that the presence of low AR activity in naïve bPSC and organoids is essential for organoid formation, which is consistent with a previous report on the indispensable role of AR during basal to luminal differentiation using conditional AR knockout mouse models^8^. Of note, inflamed bPSC were able to form a significantly larger number of organoids compared to naïve bPSC in the presence of Enz, suggesting that inflamed bPSC are more resistant to Enz treatment, likely due to their higher nuclear AR activity (Fig. 1G and 4C). In addition to reduced organoid number, Enz-treated inflamed organoids showed a significant reduction in size (Fig. 2B). Histopathological assessment further revealed an altered organoid morphology, with Enz-treated inflamed organoids more closely resembling naïve organoids. Organization and cellular stratification present in the inflamed organoids were reduced with Enz treatment, as reflected by the decreased percentage of tubule-like organoids with hollow lumens (Fig. 2C,D). Immunofluorescence staining verified the enhanced luminal differentiation in inflamed organoids with distinct central Cytokeratin 8 (CK8) (luminal epithelial marker) positive layers and peripheral CK5 (basal epithelial marker) positive layers (Fig. 2E). Enz treatment resulted in more solid organoids composed of CK8 and CK5 double positive cells (Fig. 2C,D,E), indicating that Enz blocked the complete differentiation into CK8+, CK5− luminal cells and trapped the stem cells in a transitional state. In addition to treatments with pharmacological AR inhibitors, bPSC were also isolated from *Ar*_flox/y mice and infected with lentivirus encoding Cre recombinase. The consequent knockout of AR resulted in significantly reduced number of organoids (Fig. 4F, control and AR_floxed groups). Furthermore, based on a protocol described by Henry *et al.*^16^, we enriched PSC from human prostate samples with flow cytometry as CD45−, EpCAM+, CD26− and CD49f+ cells. Treatment with Enz significantly decreased human organoid formation by 84% (Fig. 2F). Taken together, these results from the inflamed bPSC-derived organoids with manipulated AR established the underlying role of AR in inflammation-driven basal stem cell proliferation and differentiation.

### IL-1RA is upregulated in bPSC from inflamed prostates and regulates AR activity and differentiation in bPSC-derived organoids

**Figure 3.**
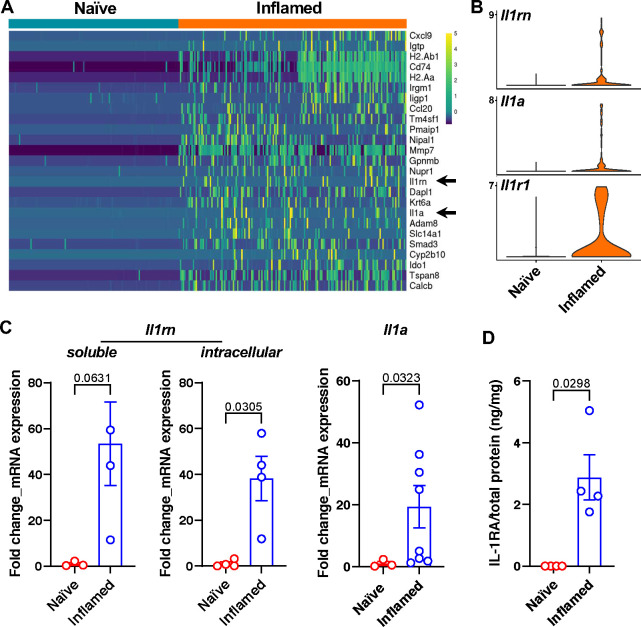
IL-1RA is upregulated in inflamed bPSC. (**A**) *Il1rn* and *Il1a* (arrows) are among the most upregulated genes in the inflamed bPSC compared to naïve bPSC as analyzed with scRNA-seq. (**B**) Violin plots of *Il1rn, Il1a* and *Il1r1* expression in naïve and inflamed bPSC. (**C**) qRT-PCR of *Il1rn* isoforms and *Il1a* in naïve and inflamed bPSC (Naïve, n=3–4; Inflamed, n=3–8). (**D**) Upregulation of IL-1RA protein is confirmed using ELISA with lysates collected from naïve or inflamed bPSC (Naïve or Inflamed, n=4).

We next investigated the mechanisms that underlie the inflammation-driven changes in the basal stem cells, including the enhanced AR activity and differentiation, by conducting a single-cell RNA sequencing (scRNA-seq) analysis comparing freshly sorted naïve and inflamed bPSC. Along with a multitude of intriguing gene expression changes (Supplemental Table 3), IL-1 receptor antagonist (IL-1RA, gene name *Il1rn*) and IL-1α (gene name *Il1a*) were identified among the top differentially expressed genes between naïve and inflamed bPSC (Fig. 3A, arrows, and Fig. 3B), whereas IL-1β was not detectable in either naïve or inflamed bPSC. IL-1 receptor 1 (IL-1R1, gene name *Il1r1*) was also identified as significantly upregulated in inflamed bPSC by scRNA-seq analysis (Fig. 3B). The upregulation of IL-1RA (both the soluble and intracellular isoforms) and IL-1α was also confirmed with qRT-PCR (Fig. 3C). Consistent with its transcript expression, IL-1RA protein could be detected using ELISA in the lysates from inflamed bPSC but was undetectable in naïve bPSC lysates (Fig. 3D). IL-1α protein was not detectable by ELISA in the lysates of either naïve or inflamed bPSC, possibly because most of IL-1α was released by the bPSC.

**Figure 4.**
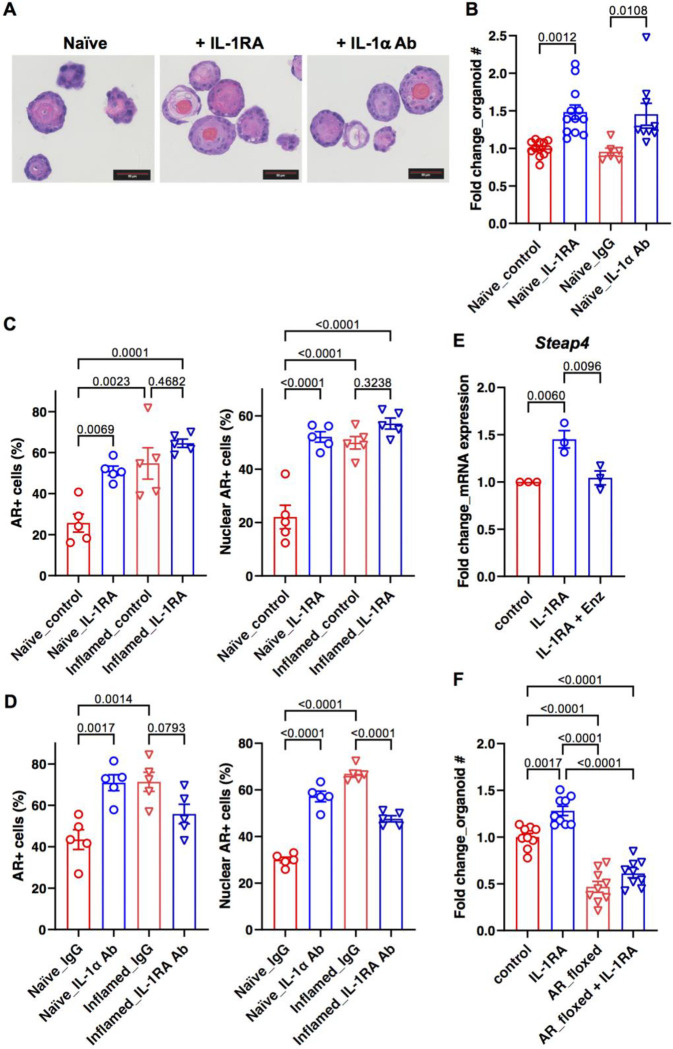
IL-1RA mediates inflammation-induced differentiation and AR activity in bPSC organoids. (**A**) Representative H&E images of naïve organoids treated with recombinant IL-1RA or neutralizing antibody (Ab) against IL-1α, showing an increase in stratification of treated organoids (Scale bar, 50 μm). **(B)** Organoid formation with naïve bPSC was significantly increased with recombinant IL-1RA (50 ng/mL) or IL-1α neutralizing Ab treatment (5 μg/mL) (Naïve_control, n=12; Naïve_IL-1RA, n=12; Naïve_IgG, n=6; Naïve_IL-1α Ab, n=9). **(C)** Quantitation of AR+ (both overall and nuclear) cells in naïve or inflamed organoids treated with IL-1RA (50 ng/mL) (All groups, n=5). **(D)** AR staining (overall and nuclear) was increased in naïve organoids treated with an IL-1α neutralizing Ab (5 μg/mL) and decreased in inflamed organoids treated with an IL-1RA neutralizing Ab (5 μg/mL) (All groups, n=5). **(E)** qRT-PCR showing the AR target gene *Steap4* was upregulated with IL-1RA in naïve organoids and Enz treatment abolished the upregulation (All groups, n=3). **(F)** The effects of IL-1RA were lost with naïve organoids derived from *Ar_*flox/y bPSC which were deprived of AR (All groups, n=9).

Given the impact of IL-1 on AR expression in prostate cancer cells and its impact on prostate cancer cell proliferation^17–20^, we reasoned that IL-1RA or IL-1α may modulate AR expression in inflamed bPSC and influence organoid growth and differentiation. We first initiated studies to evaluate the impact of IL-1RA on the differentiation and AR activity in naïve organoids. Treatment of naïve bPSC cultured for organoid development with IL-1RA recombinant protein produced organoids with distinct cellular stratification, more closely resembling those from inflamed bPSC (Fig. 4A). IL-1RA treatment also induced a significant increase in the number of formed organoids (Fig. 4B). Naïve organoids treated with an IL-1α neutralizing antibody (Ab), which blocks the binding of IL-1α to its receptor, IL-1R1, and mimics the function of IL-1RA, showed more differentiated structure and increased number as well, resembling the results from IL-1RA treatment (Fig. 4A,B). Consistent with the inhibitory function of IL-1α in organoid formation, treatment with IL-1α significantly reduced the number of organoids formed with both naïve and inflamed bPSC (Supplemental Fig. 2). These data suggest that IL-1RA promotes bPSC organoid growth and differentiation by antagonizing the inhibitory effects of IL-1α.

We next assessed the impact of IL-1RA and IL-1α on AR in bPSC-derived organoids. AR levels and nuclear localization in organoids were analyzed with immunofluorescence. The number of cells with positive AR staining, and more importantly, number of cells with AR nuclear localization were significantly increased in naïve organoids treated with IL-1RA to the level comparable with inflamed organoids (Fig. 4C). Addition of IL-1RA to inflamed organoids derived from inflamed bPSC that expressed higher level of IL-1RA did not have any significant effects on AR (Fig. 4C). The effects of IL-1RA on AR levels in naïve organoids were mirrored by those induced by an IL-1α neutralizing Ab (Fig. 4D), suggesting that IL-1RA exerts its action on AR by blocking IL-1α. Consistent with IL-1RA’s effects on AR nuclear localization, *Steap4*, an AR target gene identified in organoids (Fig. 1H), was found to be significantly induced in naïve organoids with IL-1RA treatment and the upregulation was abolished in the presence of Enz (Fig. 4E). In contrast, an antibody that neutralized IL-1RA activity was observed to inhibit both total AR level and AR nuclear localization in inflamed organoids, demonstrating that IL-1RA is the driver of AR activation during inflammation (Fig. 4D). Data supporting the indispensable role of AR downstream of IL-1RA were also obtained with genetic deletion of AR in the organoids. When bPSC were isolated from *Ar*_flox/y transgenic mice and AR was removed from the organoids with Cre recombinase lentivirus infection, IL-1RA could no longer stimulate growth in AR knockout organoids (Fig. 4F). These data demonstrate that IL-1RA, which is upregulated in bPSC during inflammation, is responsible for activated AR in this stem cell population and enhanced organoid growth and differentiation.

### Inflammation in a prostatitis mouse model promotes the basal to luminal differentiation

**Figure 5.**
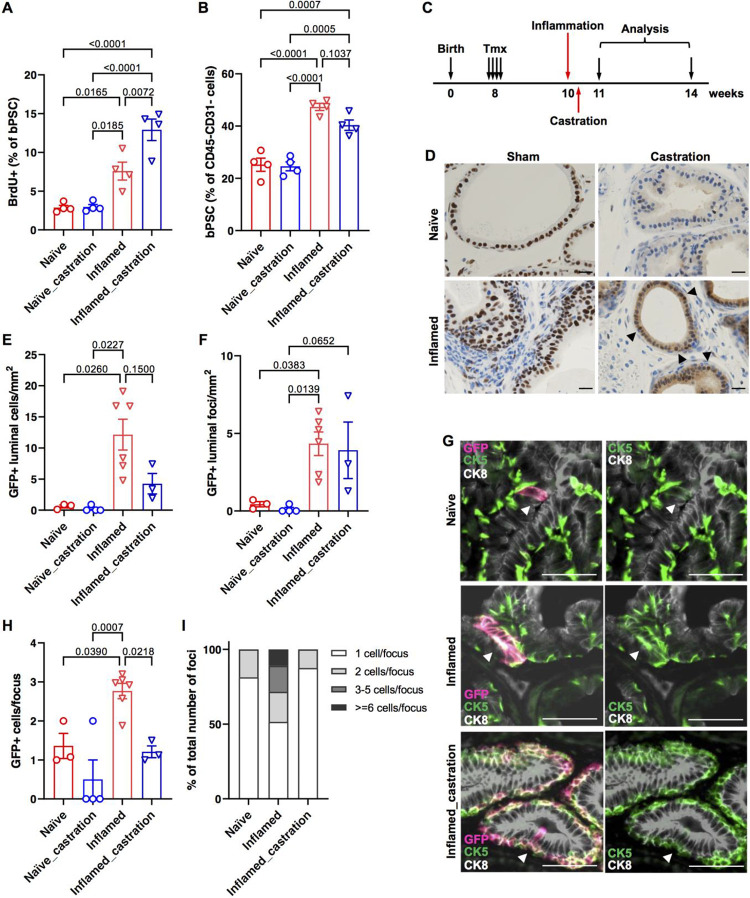
Inflammation mimicking non-bacterial prostatitis promotes the proliferation of basal stem cells and the basal to luminal differentiation *in vivo*. (**A**) Quantitation of BrdU+ bPSC in naïve and inflamed prostates, with or without castration (All groups, n=4). (**B**) Percentage of bPSC in naïve and inflamed prostates, with or without castration (All groups, n=4). (**C**) Experimental scheme showing time points of inflammation, castration and analysis. (**D**) AR staining shows maintenance of AR nuclear localization (arrow heads) in castrated inflamed prostates (Scale bar, 20 μm). (**E,F**) Quantitation of GFP+ cells or foci within the luminal layer (Naïve, n=3; Naïve_castration, n=4; Inflamed, n=6; Inflamed_castration, n=3). (**G**) Representative immunofluorescent images from naïve and inflamed prostates with or without castration showing the presence of GFP+CK5+ cells within the CK8+ luminal layer (arrowheads) (Scale bar, 50 μm). (**H,I**) Size of GFP+ foci and percentage of GFP+ foci of different sizes within the luminal layer (Naïve, n=3; Naïve_castration, n=4; Inflamed, n=6; Inflamed_castration, n=3).

We have previously reported in our POET3/OT-1 prostatitis model that prostate inflammation expanded the size of the bPSC population *in vivo*^12^. To investigate whether inflammation increases the number of bPSC by stimulating their proliferation, POET3 mice were injected with pre-activated OT-1 T cells and BrdU incorporation was analyzed 7 days later at the peak of the inflammation. Percentage of proliferating BrdU+ cells in the bPSC population was found to be significantly increased in inflamed prostates compared to naïve prostates (Fig. 5A).

In addition to having a larger population size, we have also shown previously that bPSC isolated from inflamed POET3 mice exhibited enhanced growth and differentiation in organoid cultures, as reflected by increased organoid number, larger organoid size and more distinct differentiation of the CK8+ luminal layer and CK5+ basal layer within the organoids^12^. To follow the basal to luminal differentiation upon inflammation *in vivo* and verify the results from the *in vitro* organoid cultures, we established a lineage tracing model, by crossing the POET3 mice with the mTmG;KRT5-creERT2 mice in which the Tomato protein expressed in basal epithelial cells switched to green fluorescent protein (GFP) upon the tamoxifen (Tmx)-induced *Krt5* promoter-directed expression of Cre recombinase^21^. Lineage tracing was initiated in the resulting transgenic colony (POET3^*het*^;mTmG^*het*^;KRT5-creERT2^*het*^) with intraperitoneal injections of Tmx, then activated OT-1 T cells were injected intravenously two weeks later to induce inflammation in the prostates. Animals were euthanized a month after inflammation initiation for both histological and flow cytometry analysis (Fig. 5C). Tmx injection induced GFP expression in approximately 90% of prostate basal epithelial cells as demonstrated by immunofluorescent staining with both anti-GFP and -CK5 antibodies (Supplemental Fig. 3A). Induction of GFP in basal epithelial cells was also confirmed with flow cytometry (Supplemental Fig. 3B). To assess the extent of basal to luminal differentiation after inflammation, prostate sections were stained with antibodies against GFP, CK8 and CK5. GFP+ luminal cells were counted in anterior lobes. We focused on anterior lobes because of their relative larger area, even under castrate conditions as described later. Total number of GFP+ cells found within the CK8+ luminal layer (GFP+CK8+) per mm^2^ of acinar area was dramatically increased in inflamed prostates (Fig. 5E). Increased number of GFP+ luminal cells in inflamed prostates was not only due to increased basal to luminal differentiation, as reflected by the number of GFP+ foci in the luminal layer (Fig. 5F), but also due to increased proliferation of GFP+ cells after they differentiated into luminal cells, as reflected by the number of cells within each GFP+ focus (Fig. 5G,H). Most (81%) GFP+ luminal cells found in naïve prostates were singles and the rest of the GFP+ luminal foci contained at most two cells (Fig. 5G,I), whereas GFP+ luminal cells found in inflamed prostates clustered in groups averaging 2.8 cells/cluster (Fig. 5G,H). 17% and 11% of GFP+ luminal foci in inflamed prostates were found to contain 3–5 cells and over 6 cells, respectively (Fig. 5I). Increased number of luminal GFP+ cells (identified as EpCAM+/CD49f^low^/GFP+) in inflamed prostates were also confirmed with flow cytometry (Supplemental Fig. 3C). Of note, nearly all of the GFP+ luminal cells expressed both luminal and basal markers (positive for both CK8 and CK5) (Fig.5G) in both naïve and inflamed prostates, suggesting luminal cells derived from basal stem cells retained basal features and were of a transitional phenotype, at least at the one-month time point. Taken together, data collected from our lineage tracing model, together with those from the BrdU incorporation experiment and organoid cultures, demonstrate that inflammation promotes bPSC proliferation, their basal to luminal differentiation and proliferation of the luminal epithelial cells.

Given that inflammation modulates AR level and activity in bPSC, we next examined whether inflammation, by boosting AR signaling independent of androgen, is able to promote bPSC expansion and their differentiation into luminal cells *in vivo* under castrate conditions. Castration was performed two days after the initiation of inflammation so that the inflammation was accompanied with reducing levels of androgen. BrdU incorporation was analyzed 7 days after the initiation of inflammation and the presence of GFP+ luminal cells was analyzed one month later (Fig. 5C). We first examined AR intensity and nuclear localization with immunohistochemistry to confirm diminished AR activity after castration. AR nuclear staining in the epithelial cells was found to be more intensified in inflamed prostates one week after the initiation of inflammation compared to naïve prostates. A faint, diffuse cytoplasmic AR staining pattern with little nuclear localization was observed in naïve prostates at merely five days after castration, indicating a sharp reduction in circulating androgen, whereas the nuclear AR staining persisted in inflamed prostates in castrated mice (Fig. 5D). Stronger cytoplasmic AR staining was also observed in castrated inflamed prostates.

Inflammation-induced expansion of bPSC persisted with castration (Fig. 5B) and bPSC proliferation as shown by BrdU incorporation remained significantly increased in inflamed prostates even after castration (Fig. 5A), consistent with our previous findings that bPSC population size in inflamed prostates remained unaffected with castration conducted two weeks before inflammation^12^. Interestingly, castration further increased the percentage of proliferating bPSC in inflamed prostates, suggesting a synergistic effect of inflammation and androgen deprivation on bPSC proliferation (Fig. 5A). Flow cytometry analysis of CD45+ leukocytes infiltrated into the prostates confirmed that robust inflammation was induced under the castrate condition (Supplemental Fig. 4). In our lineage tracing model, neither total number of GFP+ luminal cells or number of GFP+ foci was significantly altered with castration in inflamed prostates (Fig. 5E,F). However, the size of GFP+ foci within the luminal layer in inflamed prostates was significantly reduced with castration (Fig. 5H), with 88% of GFP+ luminal foci containing only one cell, similar to naïve prostates (Fig. 5G,I). These data demonstrate that the reduced level of androgen during inflammation does not affect the proliferation of bPSC, nor does it affect their basal to luminal differentiation. However, normal levels of androgen are essential for the proliferation of luminal cells during inflammation, as the transdifferentiated GFP+ cells stopped growing into multi-cell clusters with castration.

**Figure 6.**
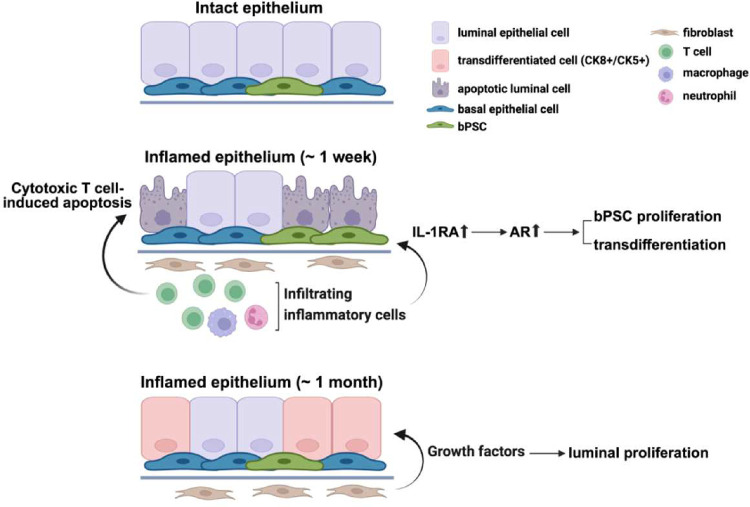
Diagram of enhanced AR signaling, bPSC proliferation and differentiation that are mediated through IL-1RA during prostate inflammation that mimics the non-bacterial prostatitis. (This image was made at Biorender.com)

In summary, we have demonstrated in a prostatitis model that AR activity is stimulated in basal stem cells by inflammation and is essential for the luminal differentiation of these stem cells. IL-1RA, which is strongly upregulated during inflammation, was identified for the first time as the driver for AR activation independently of androgen in these stem cells and consequently responsible for inflammation-induced differentiation (Fig. 6). Given the identical results from IL-1RA or IL-1α neutralizing Ab treatments, IL-1RA may trigger the downstream pathway that leads to AR activation by antagonizing IL-1α. Furthermore, we have demonstrated that inflammation, by stimulating AR signaling in an androgen-independent fashion, promotes the proliferation and differentiation of bPSC, even under castrate conditions.

## Discussion

Although strong correlations have been reported between prostate inflammation and BPH risk and progression^1–4^, how inflammation contributes to the etiology of BPH remains poorly understood. Data reported herein identify a novel mechanism, in which inflammation drives the proliferation and differentiation of the stem cell population within the basal epithelial layer, by inducing IL-1RA, which results in the enhancement of AR activation (Fig. 6). The proposed mechanism was supported by data from both *in vitro* organoid cultures and an *in vivo* lineage tracing model. Using organoid cultures for mechanistic studies, we have demonstrated that IL-1RA is not only sufficient to induce AR activation independently of androgen and promote differentiation in naïve bPSC-derived organoids, but also an essential mediator in inflammation-driven AR activation, as inhibition of IL-1RA abrogated AR activation in inflamed bPSC-derived organoids. In our lineage tracing model, inflammation greatly promoted the basal to luminal differentiation even under castrate conditions by stimulating AR in bPSC independently of androgen. Furthermore, we have demonstrated that by eliminating AR through both pharmacological inhibition and genetic deletion, differentiation of bPSC into the luminal lineage was significantly inhibited in organoid cultures, which corroborates a previous report on the indispensable role of AR in the basal to luminal differentiation^8^ and further suggests that inflammation is an important contributor to epithelial hyperplasia through the bPSC to luminal differentiation process. Further studies are necessary to better understand the biological implications of basal to luminal differentiation relative to homeostatic maintenance via luminal progenitors^9, 22^.

In our inflammation model, while castration did not impact inflammation-induced basal to luminal differentiation, it inhibited the proliferation of differentiated luminal cells. Luminal GFP+ cells differentiated from the basal layer remained as single cells, in contrast to the multi-cell luminal GFP+ clusters observed in non-castrate inflamed prostates. These data suggest that inflammation may not affect AR activity in fibroblasts where AR activation induces fibroblast secretion of growth factors such as NRG, IGF1 or FGF10. These growth factors bind to their corresponding receptors on luminal epithelial cells and are critical for luminal proliferation and replenishment during the prostate castration and regeneration cycle^9^ and in non-cell autonomous prostate growth in sub-renal capsule simulated prostate growth^23^. Under castrate conditions, inflammation is not sufficient to sustain AR in fibroblasts and provide paracrine signals for luminal proliferation.

IL-1RA exists as both a secreted form (sIL-1RA) and multiple intracellular non-secreted forms^24, 25^. The soluble extracellular sIL-1RA acts as an antagonist for IL-1R1 and competes with IL-1α or IL-1β for IL-1R1 binding. The intracellular IL-1RAs have been shown to inhibit IL-1R1-mediated target gene transcription but without affecting surface IL-1R1 engagement^26, 27^. Although IL-1RA (either the precursor of the soluble isoform or intracellular isoforms) could be easily detected in the lysates of inflamed bPSC (Fig. 3D), consistent with the dramatic upregulation of *Il1rn* transcripts with inflammation as revealed by our scRNA-seq analysis and qRT-PCR, it is more likely that the extracellular soluble form mediates the effects of inflammation. First of all, enhanced differentiation and AR activation observed in inflamed organoids could be recapitulated in naïve organoids with the addition of recombinant IL-1RA in the culture media. Conversely, the aforementioned phenotypes of inflamed organoids could be reversed with the addition of a neutralizing antibody against IL-1RA. Secondly, the effects of recombinant IL-1RA resemble those from an IL-1α neutralizing antibody, suggesting that sIL-1RA functions by antagonizing IL-1v extracellularly and relieving IL-1α’s suppression of AR activity^18–20^. IL-1R1 is likely to be the link connecting IL-1RA with the subsequent intracellular events as our previous findings have demonstrated with a bacteria-induced prostate inflammation model that inflammation-induced expansion of basal stem cell population was significantly reduced in IL-1R1 knockout mice^14^. *In vivo* infiltrating leukocytes such as myeloid cells in the inflamed prostates might also be the source for sIL-1RA^28, 29^. The expression pattern of IL-1RA in inflamed prostates, how IL-1RA from different sources contributes to stem cell AR activation and differentiation, and other inflammatory factors^30–32^ that may play critical functions in AR stabilization during prostate hyperplasia are currently under investigation.

In contrast to the presence of AR in luminal prostate cells and some mature basal cells, it has been reported by Xin *et al.* that only degraded AR was present in freshly isolated naïve bPSC and in organoid cultures derived from them^15^. Our data have shown the presence of full-length AR in the lysates from both inflamed bPSC and organoids, suggesting that inflammation may promote the stabilization of AR (Fig. 1C,F). Inflammatory signals such as cytokines and growth factors have been observed in the prostate cancer setting to stabilize AR independent of androgen through phosphorylation events^33–37^. It is possible that IL-1RA may bind to IL-1R, modulating NFκB pathway members^20^, altering the phosphorylation status of AR and ultimately leading to AR stabilization. Understanding the mechanisms connecting inflammation, IL-1RA and AR will potentially benefit the treatment of not only the benign prostate diseases such as BPH but also malignant castration-resistant prostate cancer.

Taken together, our studies have identified IL-1RA as a novel AR modulator in the inflamed prostate. IL-1RA is both sufficient and necessary for the stimulation of AR expression and activity in bPSC and may be critical in inflammation-induced bPSC expansion and differentiation and prostate hyperplasia.

## Methods

### Animal studies

Male mice aged 8–12 weeks were utilized for all studies. All animals were housed and maintained under pathogen-free conditions with 12 hour-light/dark cycles. All procedures were performed in accordance with protocols approved by Purdue University Animal Care and Use Committee (PACUC). Mice were euthanized prior to harvest by CO_2_ asphyxiation followed by secondary cervical dislocation.

POET3 mice (C57BL/6 background) were generated as previous described^10^. Mice were maintained in homozygous colonies. Inflammation was induced by adoptive transfer of 5×10^6^ pre-activated OT-1 cells (described below) via retro-orbital injection. Inflammation was allowed to develop for 7 days before prostate harvest for bPSC analysis and organoid culture. For inflammation/castration studies, mice were inflamed by adoptive transfer two days before surgical orchiectomy. BrdU labeling and analysis were performed according to instructions provided with APC BrdU Flow Kit (Becton Dickinson). Mice were injected with BrdU intraperitoneally 2 hours before euthanasia.

OT-1 mice were purchased from Jackson Laboratories (Strain #:003831). As previously described^10, 12^, spleens from OT-1 mice were ground between frosted slides in RPMI-1640 medium (Gibco) with 10% fetal bovine serum (Corning). The resulting cell slurry was filtered through a 70 μm cell strainer before treatment with Ammonium-Chloride-Potassium (ACK) lysis buffer to remove red blood cells. Remaining splenocytes were re-suspended in RPMI-1640 medium and plated at 1×10^6^/well in 24-well plates with 1:1000 beta-mercaptoethanol (Gibco) and 0.2 μg/mL SIINFEKL peptide (Ova peptide 257–264, AnaSpec). Cells were activated for 48 hours before purification using Ficoll (Cytiva) according to manufacturer’s protocol.

For lineage tracing studies, POET3 mice were crossed with mTmG mice (Strain #:007676, Jackson) and KRT5-CreERT2 mice (Strain #:029155, Jackson) to generate POET3^*het*^;mTmG^*het*^;KRT5-creERT2^*het*^ mice that were used in the studies. To initiate lineage tracing, mice were injected intraperitoneally with tamoxifen (Tmx) (Sigma-Aldrich) dissolved in corn oil at the dosage of 3 mg/40 g (body weight) for four consecutive days. Inflammation was induced via injection of pre-activated OT-1 T cells 2 weeks after Tmx injection and mice were euthanized for analysis one month after inflammation.

*Ar*_flox/y mice strain was a generous gift from Dr. Robert E. Braun at The Jackson Laboratories. AR deletion was achieved with Cre-GFP lentivirus (SignaGen Laboratories) infection. The protocol of lentiviral infection was adopted and modified from Shahi *et al.*^38^. Briefly, 10^5^ bPSC isolated from *Ar*_flox/y mice were mixed with 100 μL lentivirus and 4 μg/mL polybrene and centrifuged at 1,800 rpm for 90 minutes. Cells were washed once with DMEM media (Gibco) and re-suspended for organoid culture as described below.

Orchiectomy: Mice were anesthetized using isoflurane gas. After thorough cleaning of the surgical area, a small incision was made at the base of the scrotum. Another, smaller incision was made in the inner membrane surrounding the testicle, which was pushed out by gentle pressure on the abdomen. Once extracted, the connective tissue and blood vessels were cauterized with heated forceps before cutting. This was repeated for the second testicle. The incisions were sutured shut. Flunixin and Bupivacaine were administered as analgesics.

### Isolation of bPSC population

This process was a modification of a well-established protocol^12, 39^. Briefly, minced prostate tissues were digested in 1 mg/mL collagenase (Sigma-Aldrich) in RPMI-1640 (Gibco) media containing 10% FBS (Corning) with shaking at 37°C for 2 hours, followed by trypsinization. Dissociated cells were passed through 20G needles and 40 μm cell strainers to eliminate aggregates, followed by removal of red blood cells by ACK buffer. To enrich murine bPSC, isolated cells were stained with Zombie Violet Live/Dead Fixable Viability Dye (Biolegend) in PBS, followed by incubation with 1:100 diluted fluorescence-conjugated specific antibodies (Biolegend): CD45-FITC (#103108), CD31-FITC (#102506), Sca-1-APC (#122512) and CD49f-PE (#313612). Human prostate samples from unidentified patients were acquired from Indiana University School of Medicine Tissue Repository under IRB-approved protocols. Isolated human prostate cells were stained with Zombie UV Fixable Viability Dye (Biolegend), CD45-FITC (#304006), EpCAM-PE (#324206), CD26-APC (#302710) and CD49f-BV421 (#313624) as described in detail by Strand *et al.*^40^. Once stained, fluorescence activated cell sorting was performed on the BD FACSAria under sterile conditions.

### Organoid culture

Organoid culture protocol was adapted from Lukacs *et al*.^39^. Briefly, sorted bPSC were counted and resuspended in a 1:2 mixture of Prostate Epithelial Growth Medium (PrEGM, Lonza) and Matrigel (growth factor reduced, Corning). The mixture was deposited in a ring around the edge of a refrigerated 12-well plate at 10^4^ cells/120 μL/well and allowed to solidify for 15 minutes at 37°C before addition of pre-warmed PrEGM with or without treatments. Growth medium with treatments was changed every 2–3 days before counting and harvesting on day 7. The following treatments were used with vehicle controls: Enzalutamide (Selleckchem), R1881 (Sigma-Aldrich), recombinant mouse IL-1RA (R&D Systems), recombinant mouse IL-1α (R&D), IL1-RA neutralizing antibody (#AF-480-NA, R&D) and IL-1α neutralizing antibody (#AF-400-NA, R&D). Intact organoids were released from Matrigel with Dispase (Gibco), fixed in 10% neutral buffered formalin followed by 70% ethanol, and embedded in HistoGel (ThermoFisher Scientific).

### AR target gene PCR array

Total RNA was isolated from freshly sorted bPSC or organoids using TRIzol Reagent (Invitrogen) according to manufacturer’s protocol. cDNA was synthesized using M-MuLV Reverse Transcriptase (New England Biolabs). Global AR target gene array analysis was conducted with Qiagen RT2 Profiler PCR Array-Mouse Androgen Receptor Signaling Targets (#PAMM-142ZF) and Qiagen online data analyzer. Differentially expressed genes in both naïve and inflamed groups (threshold cycle <35) were further subjected to statistical analysis, where the significance was determined by α=0.05.

### Quantitative real-time polymerase chain reaction

Quantitative real-time polymerase chain reaction (qRT-PCR) was conducted using PerfeCTa qPCR FastMix II (QuantaBio) together with 6-FAM/ZEN/IBFQ primer/probe sets (PrimeTime qPCR Assays, IDT) and VIC-labeled TaqMan Ribosomal RNA Control Reagents (Applied Biosystems) on Roche LightCycler 96 according to manufacturers’ protocols. Triplicate samples from three independent mice were analyzed for each group. Relative gene expression was calculated using the formula 2^−[Ct(target gene)−Ct(18S)] where Ct refers to the cycle threshold.

### Western blot

Protein was isolated from freshly sorted bPSC or organoids using TRIzol Reagent (Invitrogen) according to manufacturer’s protocol and 30–50 μg protein lysates were used. AR protein was detected using rabbit anti-AR (N-20) antibody (#sc-816, Santa Cruz Biotechnology) at 1:200 dilution followed by IRDye 680RD goat anti-rabbit IgG antibody (Li-Cor) at 1:10,000 dilution. The blot was then incubated with mouse anti-β-Actin or mouse anti-Vinculin antibody (Sigma-Aldrich) followed by IRDye 800CW goat anti-mouse antibody (Li-Cor). Blots were imaged using Odyssey CLx Infrared Imaging system (Li-Cor).

### ELISA

Protein was isolated from freshly sorted bPSC using TRIzol Reagent (Invitrogen). IL-1RA and IL-1α in bPSC lysates were detected using Mouse IL-1ra/IL-1F3 Quantikine ELISA Kit and Mouse IL-1 alpha/IL-1F1 Quantikine ELISA Kit (R&D), respectively, according to manufacturer’s instructions.

### Flow cytometry analysis

Protocols were adopted from our previous study^12^. Prostate tissues were processed as described above for bPSC isolation and isolated cells were stained with the following fluorescence conjugated antibodies (Biolegend): CD45-PerCP (#103130), Sca-1-APC (#122512) and CD49f-PE (#313612) before fixation with 10% neutral buffered formalin. To detect AR protein, fixed samples were stained with unconjugated rabbit anti-AR (#ab52615, Abcam, 1:50) followed by secondary stain with Alexa Fluor 488 (Invitrogen, 1:500). Prostate cells isolated from lineage tracing mice were stained with the following fluorescence conjugated antibodies (Biolegend): EpCAM-PE/Cyanine7 (#18215) and CD49f-BV421(#313623). Flow cytometry analyses were performed using BD LSRFortessa.

### Histology

Formalin fixed samples were paraffin embedded and sectioned at 4 μm onto charged microscope slides. Tissues were deparaffinized through multiple changes of Xylene and 100% Ethanol. Immunohistochemistry: Anti-AR antibody (#ab133273, Abcam) was applied at 1:500 dilution for 30 minutes and the secondary antibody ImmPRESS HRP Goat anti-Rabbit (#MP-7451-50, Novus Biologicals) was also applied for 30 minutes. Following this, the DAB chromogen was applied for 5 minutes. Slides were double rinsed with Tris buffer between all steps. Slides were counterstained with hematoxylin before covered with resinous mounting media. Immunofluorescence: The primary GFP antibody (#A-11122, Invitrogen) was applied at a 1:250 dilution for 30 minutes followed by incubation with a secondary Goat anti-Rabbit antibody conjugated with Alexa Fluor 647 (Invitrogen). Slides were blocked twice again with 2.5% normal rabbit serum followed by AffiniPure Fab Goat anti-Rabbit (#111-007-003, Jackson ImmunoResearch Laboratories) at 20 μg/mL. Primary antibodies anti-CK5 (1:500) (#905501, Biolegend) and anti-CK8 (1:1000) (#904804, Biolegend) were applied for 60 minutes. The secondary antibodies Goat anti-Rabbit conjugated with Dylight 488 and Goat anti-Mouse conjugated with Alexa Fluor 555 (Invitrogen) were applied for 30 minutes. Slides received a DAPI counterstain at 1 μg/mL for 10 minutes and were covered with ProLong Gold Antifade Mountant (Invitrogen). Slide digitization was conducted on the Leica Aperio VERSA 8 Whole Slide Scanner in the appropriate brightfield and fluorescent settings. Digital images were uploaded to eSlide Manager for analysis. For AR immunofluorescent staining, sections were incubated with the indicated primary antibodies overnight at 4°C: rat anti-AR (#MA1–150, Invitrogen, 4 μg/mL) or rabbit anti-AR (#PA5–16363, Invitrogen, 2 μg/mL). Species-specific Alexa 488 and Alexa 594-conjugated secondary antibodies (Invitrogen) were applied for 1 hour at room temperature at a dilution of 1:200. Nuclei were stained by incubation with Hoechst 33258 nuclear stain (Sigma-Aldrich) at a concentration of 1 μg/mL. All specimens were visualized using immunofluorescence intensity with the Leica 6000 epifluorescence/confocal microscope.

### Single Cell RNA Sequencing

Data was collected at the Purdue Cell Cytometry Facility using the C1 Fluidigm instrument with SMARTer chemistry (Clontech) to generate cDNA from captured single cells. The Purdue Genomics Facility prepared libraries using a Nextera kit (Illumina). Single-end 1×50 bp reads were sequenced using the HiSeq2500 on rapid run mode. FastX-Toolkit v. 0.0.13.2 (Gordon, A., FastX-Toolkit. 2009) quality trimmer was used to further trim reads based on quality score and FASTX-Toolkit quality chart was used to make read per-base quality plots. A trimscore and a trim length of 30 were used. Tophat2 was used to align reads to the *Mus musculus* GRCm38.p6 reference genome^41, 42^. Tophat2 was run with defaults except that the number of mismatches allowed was 1. The htseq-count script in HTSeq v.0.6.1^43^ and Biopython v.2.7.3 were used to generate a counts matrix in “intersection-nonempty” mode, with feature set to “exon” and attribute parameter set to “gene_id”. R version R-3.4.1 and the package Seurat v. 1.4 was used to cluster cells into subpopulations^44^ using the first 4 principal components, a resolution of 0.5, and k.param = 4. The FindAllMarkers() function within Seurat was used to identify cluster markers using the zero-inflated “bimod” model and a false discovery rate (FDR) of 0.01. FindAllMarkers() was likewise used to identify differentially expressed genes between inflamed and naïve cells, controlling the FDR at 5%. Ingenuity Pathway Analysis (IPA, Qiagen) was used to perform pathway analyses and enrichment analyses.

### Statistical Analysis

Statistical analyses were performed using GraphPad Prism (v.9.5.1). Two-tailed unpaired Student’s t-test or Welch’s t test was performed for comparisons between two groups. Unpaired ordinary one-way analysis of variance (ANOVA), or Brown-Forsythe and Welch ANOVA tests, followed by multiple comparisons was performed for comparisons among three or more groups. *P* values less than 0.05 are considered as significant. Data are presented as means ± SEM (standard error of mean).

## Data Availability

The authors declare that all data supporting the findings of this study are available within the paper (and its supplementary information files).
